# Refeeding Syndrome Complicated by Wernicke’s Encephalopathy Following Severe Hyperemesis Gravidarum and Medical Management of Miscarriage: A Case Report

**DOI:** 10.7759/cureus.110587

**Published:** 2026-06-10

**Authors:** Wisdom A Amoo, Marufa Sultana, Miroslava Slavska

**Affiliations:** 1 Obstetrics and Gynaecology, Aneurin Bevan University Health Board, Newport, GBR

**Keywords:** hyperemesis gravidarum (hg), non-alcoholic wernicke's encephalopathy, refeeding syndrome (rs), thiamine or vitamin b1 deficiency, wernicke encephalopathy

## Abstract

Hyperemesis gravidarum (HG) is a severe form of nausea and vomiting in pregnancy that may result in dehydration, malnutrition, and electrolyte imbalance. Wernicke’s encephalopathy (WE), caused by thiamine deficiency, and refeeding syndrome (RFS), a metabolic complication of nutritional rehabilitation, are rare but serious sequelae of prolonged starvation in HG. We present the case of a multiparous woman in her 30s with severe HG and missed miscarriage at approximately 16 weeks, who developed refractory hypokalaemia, hypomagnesaemia, and evolving neurological symptoms following medical management of miscarriage. Magnetic resonance imaging demonstrated symmetrical medial thalamic and mammillary body changes suspicious for WE. She improved following intravenous thiamine and vitamin B complex replacement, potassium and magnesium correction, and multidisciplinary care. This case underscores the importance of early thiamine prophylaxis in severe HG and highlights persistent refractory electrolyte abnormalities as a potential early indicator of RFS and evolving WE.

## Introduction

Hyperemesis gravidarum (HG) affects approximately 0.3%-3% of pregnancies and represents the severe end of the spectrum of nausea and vomiting in pregnancy, potentially leading to significant maternal morbidity through dehydration, malnutrition, and electrolyte imbalance [1,2]. Prolonged vomiting and poor nutritional intake predispose patients to vitamin deficiencies, particularly thiamine (vitamin B1), resulting in Wernicke’s encephalopathy (WE), an acute neuropsychiatric syndrome associated with considerable morbidity if unrecognised [3,4].

Refeeding syndrome (RFS) may also occur following nutritional rehabilitation in malnourished individuals and is characterised by electrolyte shifts including hypokalaemia, hypomagnesaemia, and hypophosphataemia [5,6]. Although both conditions have individually been reported in association with HG, their coexistence is uncommon [7,8].

This case was previously presented as a poster at the British Maternal and Fetal Medicine Society (BMFMS) Annual Conference, held in Leeds, United Kingdom, on March 27, 2026. Elements of this work have also been submitted/accepted as a conference abstract.

## Case presentation

A woman in her 30s, gravida 3 para 2 with no significant past medical history, presented to the Emergency Gynaecology Assessment Unit at eight weeks of gestation with severe vomiting and poor oral intake requiring same-day admission. She had intravenous rehydration and parenteral antiemetics and was discharged the same day on oral antiemetics following symptomatic improvement. She had further similar presentations at nine and 13 weeks; however, no thiamine supplementation had been given as she had not required dextrose-containing fluids. Her obstetric history included one emergency lower-segment caesarean section and one term vaginal delivery. Surgical history was notable for a previous cholecystectomy.

At approximately 16 weeks of gestation, a private ultrasound demonstrated absent foetal cardiac activity. Repeat imaging at the Early Pregnancy Unit confirmed a missed miscarriage. Following counselling, she underwent inpatient medical management according to local board protocol with mifepristone, followed 48 hours later by misoprostol.

On admission for misoprostol, she was tachycardic (140-160 beats per minute), normotensive (120/72 mmHg), and apyrexial (36.5°C) with electrocardiography confirming sinus tachycardia. Following delivery of the foetus, manual evacuation of retained placenta and products of conception was acquired at the bedside, with estimated blood loss of approximately 400 mL. Subsequently, she became pyrexial (39.5°C) with tachycardia (155 beats per minute), elevated inflammatory markers (C-reactive protein (CRP) 41 mg/L), and lactate of 3.9 mmol/L, raising suspicion of septic miscarriage. Initial blood investigations also demonstrated moderate hypokalaemia (2.7 mmol/L).

She was managed with empirical intravenous antibiotics and intravenous potassium chloride replacement. Although inflammatory markers improved clinically, hypokalaemia persisted (2.9 mmol/L) after 48 hours despite adequate potassium replacement. Antibiotic therapy was subsequently stepped down to oral treatment.

Medical review was sought due to electrolyte derangement as further investigations revealed hypomagnesaemia (0.57 mmol/L), prompting intravenous magnesium replacement. Additional investigations (calcium, alkaline phosphatase, and phosphate) were also requested to exclude secondary causes of refractory hypokalaemia, which were unremarkable.

Despite advice to remain inpatient, the patient self-discharged on Day 4 against medical advice with oral potassium supplementation (Sando-K). Repeat electrolyte testing 48 hours later demonstrated persistent moderate hypokalaemia (2.7 mmol/L), necessitating readmission. At this stage, worsening dizziness, fatigue, and neurological symptoms prompted a formal medical review. A cumulative total of approximately 240 mmol potassium chloride had been administered. Bone profile and repeat magnesium levels were within normal limits.

Neurological examination demonstrated variable ptosis, coarse horizontal nystagmus, right abducens nerve (cranial nerve VI) paresis, and worsening diplopia. Additional examination findings included past pointing and lower-limb hyporeflexia. Given the history of prolonged starvation secondary to severe HG, absence of thiamine supplementation, and persistent electrolyte derangement despite replacement, probable RFS with thiamine deficiency and early WE was suspected.

Computed tomography of the head was unremarkable, and a venogram ruled out a dural venous sinus thrombosis. Subsequent specialist neurological assessment confirmed bilateral horizontal gaze nystagmus and global hyporeflexia, with otherwise preserved tone, power, and muscle bulk. Magnetic resonance imaging (MRI) of the brain demonstrated subtle symmetrical T2/fluid-attenuated inversion recovery (FLAIR) and diffusion-weighted hyperintensity involving the bilateral medial thalami (Figure 1), extending adjacent to the third ventricle and likely involving the mammillary bodies (Figure 2), findings considered highly suggestive of WE in the clinical context.

**Figure 1:**
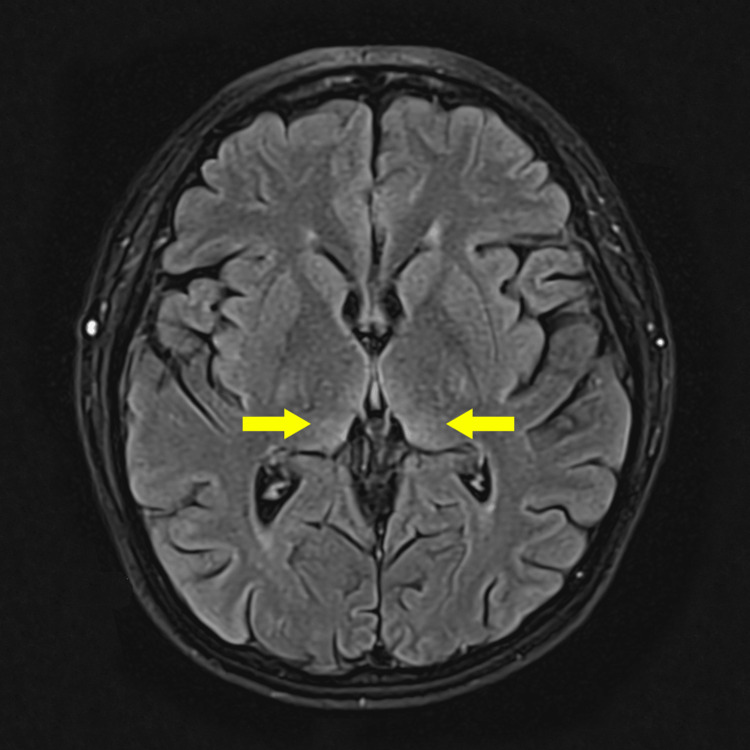
Brain MRI T2-weighted and FLAIR image sequence in the axial plane. The yellow arrows point to the areas of bilateral medial thalamic hyperintensity FLAIR: fluid-attenuated inversion recovery; MRI: magnetic resonance imaging

**Figure 2:**
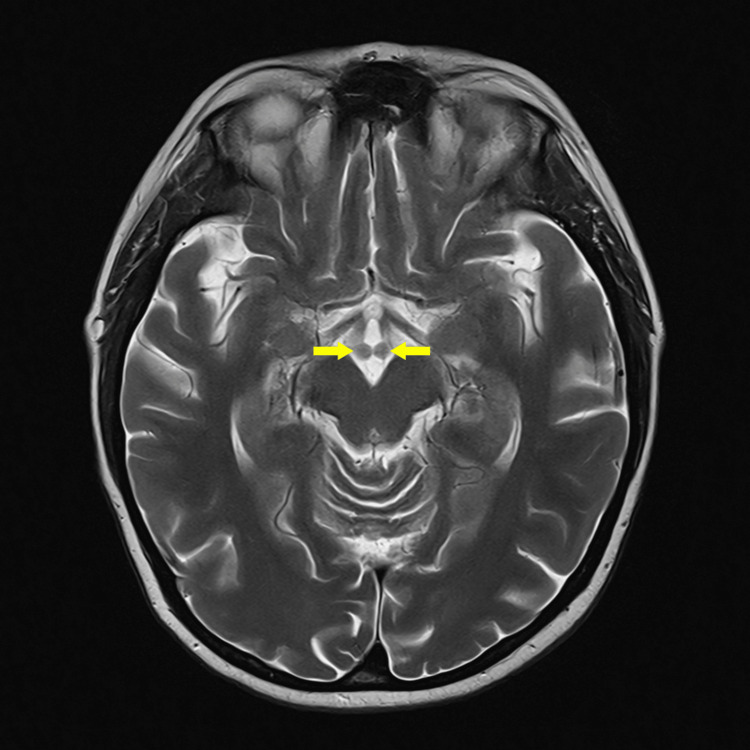
Brain MRI T2-weighted and FLAIR image sequence in the axial plane. The yellow arrows point to the areas of mammillary body involvement FLAIR: fluid-attenuated inversion recovery; MRI: magnetic resonance imaging

The patient was commenced on intravenous vitamin B and C complex (Pabrinex®), intravenous thiamine, continued potassium and magnesium replacement, and nutritional optimisation. Further nutritional screening revealed normal vitamin B12 levels of 434 pg/mL and folate deficiency (1.6 ng/mL). Daily monitoring of refeeding bloods was undertaken, and dietetic input was obtained.

The patient demonstrated progressive clinical improvement over subsequent days, with resolution of electrolyte abnormalities and neurological symptoms. Biochemical trend, therapeutic interventions, and clinical course are outlined in Table 1. She was discharged on oral thiamine, high-dose vitamin B complex with outpatient follow-up, and safety-netting advice.

**Table 1:** Serial biochemical trends, clinical progression, and therapeutic interventions during hospital admission Hb: haemoglobin; WCC: white cell count; CRP: C-reactive protein; Na⁺: sodium; K⁺: potassium; Mg²⁺: magnesium; PO₄³⁻: phosphate; IV: intravenous; KCl: potassium chloride; ECG: electrocardiogram; CT: computed tomography; MRI: magnetic resonance imaging; WE: Wernicke’s encephalopathy; NR: not recorded; GOPD: gynaecology outpatient department

Hospital day	Hb (ref: 115-165 g/L)	WCC (ref: 4.0-11.0 x 10^9^/L)	CRP (ref: <10 mg/L)	Lactate (ref: 0.5-2.2 mmol/L)	Na^+^ (ref: 133-146 mmol/L)	K^+^ (ref: 3.5-5.3 mmol/L)	Mg^2+^ (ref: 0.7-1.00 mmol/L)	PO_4_^3-^ (ref: 0.8-1.5 mmol/L)	Clinical course	Key management/intervention
Day 1 (admission for misoprostol)	121	10.6	41	3.9	136	2.7	NR	0.93	Clinically unwell following complete miscarriage	Empirical antibiotics, IV KCl replacement
Day 2	100	6.0	44	1.6	137	2.9	0.72	NR	Partial symptomatic improvement	Repeat KCl and continue antibiotics for up to 48 hours
Day 3	97	5.6	38	1.6	139	2.7	0.57	NR	Clinically stable	Medical review; Mg²⁺ assessment and replacement initiated
Day 4	89	4.5	NR	NR	139	2.7	0.59	NR	Persistent fatigue with stable observations	Discharged against medical advice, home with Sando-K, repeat electrolytes in 48 hours
Day 6 (readmission)	NR	NR	NR	NR	142	2.7	0.68	0.99	Dizziness, increasing weakness, normal ECG. Folate: 1.6 (ref: >3.0 µg/L). Vit B12: 434 (ref: 180-900 ng/L)	Readmission; electrolyte replacement, neuroimaging and neurology review
Day 7	NR	NR	NR	NR	143 (plasma)	2.9 (plasma)	0.78 (plasma)	NR	Ophthalmoplegia confirmed, weak	Neurology review; MRI findings suggestive of WE; IV Pabrinex®, thiamine and electrolyte replacement with daily refeeding bloods
Day 8	78	6.3	NR	NR	146	2.9	0.60	1.02	Improving clinical status	Dietetics review, ongoing management
Day 9	75	6.6	NR	NR	144	3.4	0.56	1.38	Clinically improving	Ongoing monitoring
Day 10	77	5.4	NR	NR	144	3.5	0.56	1.20	Clinical status unchanged	Switch to oral electrolyte supplementation, continue IV Pabrinex® and thiamine
Day 11	74	4.4	NR	NR	142	3.4	0.56	1.16	Marked clinical improvement	Ongoing electrolyte and nutritional optimisation
Day 12	84	6.4	<10	NR	143	3.6	NR	NR	Clinically stable for discharge	IV iron replacement. Discharged on oral thiamine, Vit B complex, and Forceval for 1 month
Day 20 (1-week follow-up)	92	4.3	NR	NR	140	3.9	0.78	1.07	Asymptomatic	Follow-up in 3 months
3-month GOPD follow-up	NR	NR	NR	NR	NR	NR	NR	NR	Sustained clinical recovery	Repeat MRI-resolution of previous findings

A three-month follow-up in the outpatient department noted overall clinical improvement with a repeat MRI of the brain demonstrating resolution of previously identified signal abnormalities, consistent with radiological resolution (Figures 3, 4).

**Figure 3:**
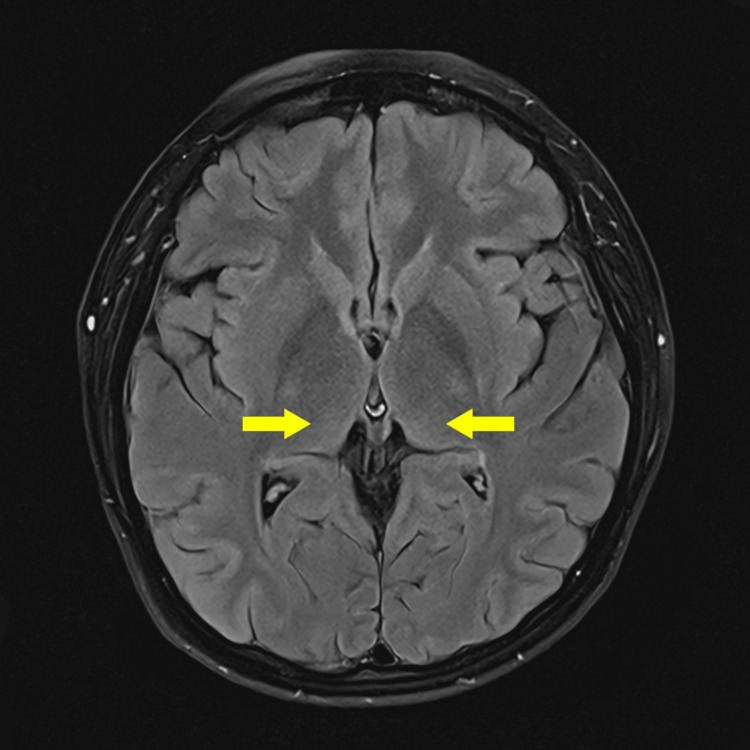
Brain MRI T2-weighted and FLAIR image sequence in the axial plane. The yellow arrows demonstrate the interval resolution of bilateral medial thalamic hyperintensity compared with Figure 1 FLAIR: fluid-attenuated inversion recovery; MRI: magnetic resonance imaging

**Figure 4:**
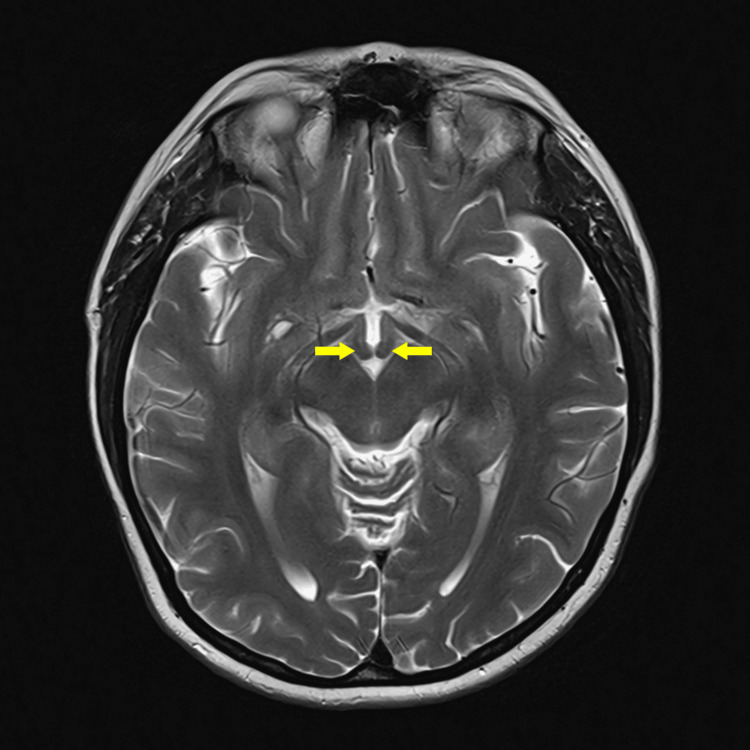
Brain MRI T2-weighted and FLAIR image sequence in the axial plane. The yellow arrows demonstrate the interval resolution of mammillary body abnormalities compared with Figure 2 FLAIR: fluid-attenuated inversion recovery; MRI: magnetic resonance imaging

## Discussion

This case illustrates a rare but clinically important coexistence of probable RFS and MRI-supported WE following severe HG and medical management of miscarriage. HG affects approximately 0.3%-3% of pregnancies and may result in dehydration, electrolyte derangement, and severe nutritional depletion [1]. Prolonged vomiting and poor oral intake rapidly deplete thiamine stores, particularly in pregnancy, where metabolic demands are increased [3]. WE, classically characterised by ophthalmoplegia, ataxia, and confusion, is increasingly recognised in non-alcoholic populations, including women with HG, and demonstrates the complete clinical triad in fewer than one-third of patients [3,8].

Our patient exhibited ocular signs including horizontal nystagmus, ptosis, diplopia, and cranial nerve VI palsy, in addition to cerebellar findings and MRI abnormalities involving the medial thalami and mammillary bodies, strongly supporting the diagnosis of WE [3,9]. The MRI findings are consistent with previously described neuroradiological patterns in WE, commonly demonstrating symmetrical signal abnormalities in the medial thalami, mammillary bodies, periaqueductal region, and tectal plate [4]. Importantly, neuroimaging should support rather than delay treatment, as WE remains primarily a clinical diagnosis requiring urgent thiamine replacement to prevent irreversible neurological injury [3].

RFS was considered a probable diagnosis despite the absence of profound hypophosphataemia. Although phosphate measurements remained largely within reference ranges, prolonged starvation, refractory hypokalaemia despite replacement, concomitant hypomagnesaemia, and the temporal relationship with nutritional rehabilitation supported the diagnosis. RFS occurs following reintroduction of nutrition after prolonged starvation and is characterised by intracellular electrolyte shifts precipitated by insulin release, resulting in hypokalaemia, hypomagnesaemia, and hypophosphataemia [7,10]. Persistent moderate hypokalaemia despite administration of approximately 240 mmol potassium chloride and concurrent hypomagnesaemia raised suspicion for an underlying nutritional-metabolic process beyond isolated gastrointestinal loss. Previous reports have described RFS complicating HG, particularly following nutritional rehabilitation or restoration of oral intake after prolonged starvation [7,10].

This case also raises an important but under-recognised clinical consideration regarding severe infection and thiamine deficiency. Following miscarriage management, the patient developed pyrexia, elevated inflammatory markers, and biochemical features suggestive of septic miscarriage. Emerging evidence suggests that severe infection and systemic inflammatory states may themselves precipitate or exacerbate thiamine deficiency through increased metabolic demand, impaired utilisation, and accelerated depletion of thiamine stores [11]. In this context, sepsis may have acted as an additional precipitating factor in the evolution of WE in an already nutritionally vulnerable patient with severe HG. To our knowledge, reports of probable RFS and WE occurring following medical management of miscarriage in a patient with antecedent severe HG remain exceedingly uncommon.

This case reinforces Royal College of Obstetricians and Gynaecologists guidance recommending prophylactic thiamine supplementation (either oral 100 mg tds or intravenous as part of vitamin B complex (Pabrinex®)) for women with prolonged vomiting, particularly before carbohydrate-containing fluids or nutritional replenishment [1]. Despite multiple healthcare encounters for severe HG, our patient had not received thiamine supplementation prior to neurological deterioration. This highlights a potentially preventable complication and emphasises the importance of maintaining a high index of suspicion in women with persistent vomiting, refractory electrolyte abnormalities, or evolving neurological symptoms [1,3].

## Conclusions

Both WE and RFS are preventable complications of severe HG. Clinicians should be vigilant in managing pregnant women with prolonged vomiting and consider further investigations for refractory electrolyte imbalance and emerging neurological signs. Early thiamine supplementation, careful electrolyte monitoring, and cautious nutritional rehabilitation following prolonged starvation are preventive measures to reduce the occurrence of WE and RFS, with the aim of reducing maternal morbidity.
